# Fractal Dimension and Texture Analysis in the Assessment of Experimental Laser-Induced Periodic Surface Structures (LIPSS) Dental Implant Surface—In Vitro Study Preliminary Report

**DOI:** 10.3390/ma15082713

**Published:** 2022-04-07

**Authors:** Jakub Hadzik, Paweł Kubasiewicz-Ross, Wojciech Simka, Tomasz Gębarowski, Ewa Barg, Aneta Cieśla-Niechwiadowicz, Anna Trzcionka Szajna, Ernest Szajna, Tomasz Gedrange, Marcin Kozakiewicz, Marzena Dominiak, Kamil Jurczyszyn

**Affiliations:** 1 Department of Dental Surgery, Faculty of Medicine and Dentistry, Medical University of Wroclaw, ul. Krakowska 26, 50-425 Wroclaw, Poland; pawel.kubasiewicz-ross@umw.edu.pl (P.K.-R.); marzena.dominiak@umw.edu.pl (M.D.); kamil.jurczyszyn@umw.edu.pl (K.J.); 2 Faculty of Chemistry, Silesian University of Technology, 44-100 Gliwice, Poland; wojciech.simka@polsl.pl; 3 Department of Biostructure and Animal Physiology, Wroclaw University of Environmental and Life Sciences, Kożuchowska 1/3, 51-631 Wroclaw, Poland; tomasz.gebarowski@upwr.edu.pl; 4 Department of Medical Science Foundation, Faculty of Pharmacy, Wroclaw Medical University, Borowska 211, 50-556 Wroclaw, Poland; ewa.barg@umw.edu.pl (E.B.); aneta.ciesla-niechwiadowicz@umw.edu.pl (A.C.-N.); 5 NanoPrime, 39-200 Dębica, Poland; ania.trzcionka-szajna@wp.pl; 6 KMB Catalyst, 43-175 Wyry, Poland; e.szajna@uce.com.pl; 7 Department of Orthodontics, TU Dresden, 01062 Dresden, Germany; tomasz.gedrange1@tu-dresden.de; 8 Department of Maxillofacial Surgery, Faculty of Military Medicine, Medical University of Lodz, 90-151 Łódź, Poland; marcin.kozakiewicz@umed.lodz.pl

**Keywords:** implant topography, fractal dimension analysis, LIPSS, laser surface, texture analysis

## Abstract

Laser-induced periodic surface structures (LIPSS) are the sub-wavelength periodic nanostructures generated by the femtosecond laser. Implant topography and its nanostructural changes can be important for biomedical applications. In order to compare the surface topography of different implants, appropriate mathematical and physical descriptive methods should be provided. The aim of the study was to evaluate the experimental LIPSS-based—Low Spatial Frequency LIPSS (LSFL) dental implant surfaces. Novel methods of surface analysis, such as Fractal Dimension Analysis and Texture Analysis, were compared to the standard surface roughness evaluation. Secondary, cell viability, and attachment tests were applied in order to evaluate the biological properties of the new titanium surface and to compare their correlation with the physical properties of the new surfaces. A Normal Human Dermal Fibroblast (NHDF) cytotoxicity test did not show an impact on the vitality of the cells. Our study has shown that the laser LIPSS implant surface modifications significantly improved the cell adhesion to the tested surfaces. We observed a strong correlation of adhesion and the growth of cells on the tested surface, with an increase in implant surface roughness with the best results for the moderately rough (2 μm) surfaces. Texture and fractal dimension analyses are promising methods to evaluate dental implants with complex geometry.

## 1. Introduction

The titanium dental implants are considered to be the gold standard of modern dental implantology. The surface of the dental implants can be considered in terms of its chemical and physical aspects (topography). The surface property of a dental implant has a decisive influence on the process of implant osseointegration that is defined as the stable anchorage of an implant achieved by direct BIC bone-to-implant contact [1,2]. After being inserted into a bone, the dental implant surface should promote bone healing and consequently the formation of well-organized bone with a high BIC [3]. The first osseointegrated surfaces of the dental implants were produced by the industrial machining of bulk titanium implants, and the development of the physical characteristics of the dental implant surfaces is responsible for the progress that has been made in recent decades in dental implantology.

The implant micro-surface and nano-surface modifications have been proven to affect cellular responses such as cell adhesion, proliferation, differentiation, and migration, thus influencing bone healing [4,5]. Due to surface modifications, it was possible to overcome the adverse effects of length reduction and the unfavorable crown–implant ratio of short implants [6,7], shortening the time needed to achieve secondary stability and deliver prosthetic restoration [8,9].

Recently, a Laser-Induced Periodic Surface Structure (LIPSS) surface of the dental implant has been in the area of interest. The term LIPSS was launched in the literature by Driel and co-workers in 1982 [10]. When the laser operates with pulse duration, a structure appears as reliefs and grooves on the titanium implant surface. LIPSS-based dental implant topography and its nanostructural changes can be important for biomedical applications.

In order to compare the surface topography of different implants, appropriate mathematical and physical descriptive methods should be provided. A scanning electron microscope (SEM) with a tungsten source is one of the standard devices to characterize morphology at the micrometer level. The geometrical specifications and surface texture are specified in the ISO 21920-2:2021 [11]. The key surface parameters for the quantitative description of the implant surface topography are Ra, Rz, and Sa. The degree of roughness marked is Ra (*average roughness)*, which is an average measure of the deviation value of the individual surface points in relation to the selected reference plane; Rz is referred to as the maximum vertical roughness; and Sa is the average surface height deviation amplitude [12]. A surface roughness of Sa values between 1 μm and 2 μm appears to be optimal [2]; however, the mechanisms behind an optimal bone response to this Sa level remain largely unknown and often also depend on the surface chemical properties.

A fractal dimension (FD) analysis is used in the estimation of complex, irregular shapes or surfaces. As the fractal architecture concept is particularly interesting in surface and material science, it can also be adapted to assess the surface of titanium endosseous implants. Fractal dimension analysis (FDA) has been reported in the surface testing of various dental materials, among them xenogenic bone, lithium disilicate-based crowns, zirconia dental implants, or dental restorative, composite, orthodontic wires [13]. Texture analysis (TA) is another mathematical method that enables the analysis of the surface. The pixels form an image structure called a texture. A texture is a set of graphic patterns which are characterized by brightness, entropy, smoothness, uniformity, roughness, granularity, randomness, or linearity. TA offers the possibility of surface analysis. Texture analysis is widely applied to magnetic resonance images, computed tomography, and X-ray images.

The aim of the study was to evaluate the experimental LIPSS-based—Low Spatial Frequency LIPSS (LSFL) dental implant surface. Novel methods of the surface analysis, such as Fractal Dimension Analysis and Texture Analysis, are compared to the standard surface roughness evaluation. The null hypothesis was that there was a correlation between increases in the FDA and TA values and implant surface roughness. For implants characterized by high FDA (higher complexity topographic feature) and TA, a greater cell adhesion will also be observed.

We put forward the following null hypotheses:

**Hypothesis 1 (H1).**  *There are no statistical differences between the examined surfaces in the aspects of FDA and TA*.

**Hypothesis 2 (H2).**  *There is no correlation between FD, TA, and Sa, the amount of fibroblasts in mm^2^, and medium au*.

## 2. Materials and Methods

For the purposes of this study, titanium plates as samples of different LIPSS-based dental implant experimental surfaces were delivered and tested. The roughness of the surface was measured. Based on the SEM images, fractal dimension analysis and texture analysis were calculated. All the plates were tested for cytotoxicity, and finally, human gingival fibroblasts were used to assess the cell culture adhesion to each sample.

### 2.1. Titanium Plates Preparation and Surface Modification

For the purposes of the study, titanium plates with a standard SLA surface (sandblasted and acid etched) and an experimental LIPSS-based surface—Low Spatial Frequency LIPSS (LSFL) were prepared by the NanoPrime company (Dębica, Poland). The LIPSS surface samples were fabricated under the production name HELT (HELT, HELT-HA) from Titanium Grade 4 (TiGr4) and Titanium Grade 23 (TiGr23). Six samples of each surface were prepared and used for the study. The details of the titanium surface samples used in the study are presented in Table 1.

### 2.2. Surface Analysis Surface Topography (Sa) Measurement

The surface roughness parameter (Sa) was studied with use of a scanning electron microscope SEM (Phenom ProX, Phenom-World BV Thermo Fisher Scientific, Waltham, MA, USA), applying 15 keV accelerating voltage. The roughness parameters were measured with use of Phenom 3D Roughness Reconstruction Software (version 1, Phenom-World BV, Thermo Fisher Scientific, Waltham, MA, USA ). Each surface was triplicated, and the measurements were performed in four places on each sample. The results were averaged, and a standard deviation (SD) was calculated.

### 2.3. Fractal Dimension Analysis

Two different magnifications of SEM images (5000× and 100,000×) for fractal dimension analysis were used. Ten SEM images were taken from each implant. From each image, 5 regions of interest (ROIs) were selected. In the case of 5000× magnification, the ROI dimension was 100 μm × 100 μm; for 100,000× magnification, 5 μm × 5 μm was set. Thus prepared, the ROIs were analyzed using a modified algorithm of the classical counting box method—the intensity difference fractal dimension counting method.

An ImageJ version 1.53e (Image Processing and Analysis in Java—Wayne Rasband and contributors, National Institutes of Health, Bethesda, MD, USA, public domain li-cense, https://imagej.nih.gov/ij/, accessed on 1 January 2022) and the FracLac plugin version 2.5 (Charles Sturt University, Bathurst, Australia, public domain license) was used to perform all fractal analysis.

This algorithm allows us to analyze 8-bit monochromatic images. It was fully de-scribed in our previous study [14]. In this method, the analyzed image is divided into boxes such as in scale ε. The image size selected for analysis was 100 × 100 μm and 5 × 5 μm. FDA consists of some repeatable steps. For example: at the first step, the grid size equals 100 μm (dimension of analyzed image, ε = 1); in the next steps, ε is divided by 2 (ε value for following steps: ε = 0.5, ε = 25). In each step, the difference of the pixels’ brightness intensity is calculated in every grid in scale ε. In FracLac software, this algorithm of the ε calculation is called a block series. This option scans a square block within an image using a series of grids calculated from the block size. This way is most usable for the analysis of the pattern which fills the whole area of the image.

The difference between the maximum pixel intensity and the minimum pixel intensity is counted in each box:

*δIi,j,ε* = *maximum pixel intensity i,j,ε* − *minimum pixel intensity i,j,*ε (1)

where *δI*—the difference between the maximum pixel intensity and the minimum pixel intensity *i*, *j*—coordinates of the analyzed box in a scale ε.

In the next step, 1 is added to the intensity difference to avoid its value becoming 0:

*Ii,j,ε* = *δIi,j,ε* + 1 (2)

Finally, the fractal dimension of the intensity difference is described by the following formula

(3) $$FD=\underset{\varepsilon \to 0}{lim}\left(\frac{lnI\left(\varepsilon \right)}{\frac{1}{\varepsilon }}\right)\;$$

where *FD*—fractal dimension of intensity difference, *I*(*ε*) = Σ[*δIi*,*j*,*ε* + 1], and *ε*—scale of box.

### 2.4. Surface Analysis TA

The texture of the SEM images was analyzed in MaZda 4.6 software, invented by the University of Technology in Lodz [15], to check how the features were describing the lesion observed. The primary 8-bit optical depth was reduced to 6 bits. The region of interest (ROI) was normalized (μ ± 3σ) to share the same average (μ) and standard deviation (SD) of optical density within the ROI. The differential entropy (DifEntrp) was selected as a texture feature from the co-occurrence matrix to calculate in the ROI:

(4) $$DifEntr=-\sum_{i=1}^{Ng}p_{x-y}\left(i\right)log\left(p_{x-y}\left(i\right)\right)$$

where Σ is sum, *Ng* is the number of levels of optical density in the radiograph, *i* and *j* are the optical density of the pixels 5 pixels distant one from another, *p* is probability, and *log* is the common logarithm [16]

### 2.5. Biological Analyses

#### 2.5.1. Cell Culture

The Normal Human Dermal Fibroblast (NHDF) cell line from Lonza (Basel, Switzerland) and the L929 cells were purchased from Sigma-Aldrich (Merck Group, Headquarters, Darmstadt, Germany) (The European Collection of Authenticated Cell Cultures—ECACC). All cells were cultured at 37 °C, in 5% CO_2_, 95% humidity in a CO_2_ incubator. The cells were cultured for a minimum of 2 weeks before the assay. If the confluence exceeded 70% (confluence measurement, JuliBr microscope, NanoEntek, Seoul, Korea), the cell cultures were passaged with trypsin/EDTA solution. The cells were counted with an automatic cell counter NucleoCounter^®^ NC-200 (ChemoMetec A/S, Allerod, Denmark). If the confluence was less than 70%, the medium was replaced with fresh medium. The cells were cultured in Dulbecco’s modified Eagle medium (DMEM) without phenol red, 10% fetal bovine serum (FBS), penicillin (10,000 U/mL), streptomycin (10 mg/mL), and L-glutamine (200 mM). All culture reagents were purchased from Biological Industries (Beit HaEmek, Israel).

#### 2.5.2. Preparation of Samples

All test samples were individually packaged and autoclaved for sterilization. For the in vitro biological cytotoxicity assessment, liquid extracts of the test materials were prepared according to the provisions of the standard: EN ISO 10993-5: Biological evaluation of medical devices—Part 5: Tests for in vitro cytotoxicity. According to these recommendations, a culture medium with serum was used for tests on mammalian cells. Extraction was performed in sterile, chemically inert, sealed tubes for 24 h at 37 °C. For the other tests, the test material was stored at room temperature after sterilization, for up to one week. Before performing the experiments involving direct assessment of the interaction of materials with cells, the prepared sections were moistened with the culture medium with serum.

#### 2.5.3. Proliferation Assay

The test was performed in accordance with the guidelines of the standard. The cell cultures were obtained from culture bottles by enzymatic digestion (trypsin/EDTA). The obtained cell suspension was centrifuged (200× *g*, 3 min). The cells were then counted and resuspended in culture medium. The density of the cell suspension was 1 × 10^5^ cells/mL. Using a multichannel pipette, the cells were dispensed at 100 µL into 96-well plates at 1 × 10^4^ cells/well). The cells were incubated for 24 h (5% CO_2_, 37 °C, 90% humidity) so that the cells would form a monolayer on the plate surface. This incubation period ensures cell regeneration, cell adhesion, and the transition to the exponential growth phase.

Before further experimentation, each plate was checked under a phase contrast microscope to ensure that the cell growth was relatively uniform across the test plate. A check was performed to identify experimental errors.

After 24 h of incubation, the medium was removed from above the cells. Then, 100 µL of medium containing the appropriate sample extracts, control or blank only, was added to each well. The test plates were incubated for a further 24 h (5% CO_2_, 37 °C, 90% humidity).

After 24 h incubation, each plate was viewed under a phase-contrast microscope to identify errors in cell seeding and to assess the growth of the control and extract-treated cells. Observed changes in cell morphology may be due to the cytotoxic effects of the test sample extract. The culture medium was then carefully removed from the plates, and 50 µL of MTT solution at a concentration of 1 mg/mL was added to each well. The plates were incubated for a further 2 h in an incubator at 37 °C. After this time, the MTT solution was removed, and 100 µL of isopropanol was added to each well. Absorbance was read on a MultiscanGo reader (Thermo Scientific, Waltham, MA, USA) at 570 nm.

#### 2.5.4. Cell Attachment

The cell cultures obtained from the culture bottles by enzymatic digestion (trypsin/EDTA) were also used for the human fibroblast adhesion assay. The obtained cell suspension was centrifuged (200× *g*, 3 min). The cells were counted and resuspended in culture medium. The density of the cell suspension for the adhesin test was 1 × 10^6^ cells/mL. Twenty-four-well plates were used for the test. The test materials were placed in the wells. The cells in the test were applied to the wetted material with an automatic pipette. After application, the cells were incubated for 2 h (5% CO_2_, 37 °C, 90% humidity) so that the cells could adhere to the test materials. After this time, the wells were supplemented with the culture medium with serum in a volume of 1000 µL. The test plates were incubated for a further 72 h (5% CO_2_, 37 °C, 90% humidity). After the incubation time, the cells growing on the test surfaces were stained using the Cell Viability Imaging Kit (Blue/Green). Staining involves adding a dye to the culture and incubating for 5 to 30 min. After this time, images were taken using a BioTek Lionheart microscope (Agilent Technologies, Santa Clara, CA, USA) using fluorescence excitation with a led illuminator: ex 377 em 447 and ex 469 em 525. Further analysis was performed using GEN5 dedicated image analysis software (Agilent Technologies, Santa Clara, CA, USA). The fluorescence intensity and the number of cells stained with each dye were analyzed. The cells showing blue fluorescence were counted as alive and those showing green as dead.

#### 2.5.5. Co-Culture of Cells with Materials

By performing this assay, the cells were prepared as in the cell-attachment method. For this assay, the cells were seeded into 24-well plates alongside previously placed test materials. In this method, the direct interaction between the cells and the test material was checked by evaluating the morphology and staining as in the previous method, but only the percentage of live and dead cells was evaluated on a BioTek Lionheart microscope. To ensure that no direct cytotoxic effects were observed, the samples in the co-culture assay were incubated for 72 h.

### 2.6. Statistical Analysis

Statistica version 13.3 (StatSoft, Cracow, Poland) was applied to calculate all the fractal dimension analysis statistical tests, and 0.05 was set as the statistically significant level. The normality of distribution was confirmed by the Shapiro–Wilk test. Due to normal distribution, we performed parametric tests. Analysis of variance (ANOVA) and post hoc least significant difference were used to show the differences in fractal dimensions between each surface in two scales. The correlation matrix was applied to calculate the Pearson correlation coefficient (r) between the fractal dimensions of the lesions in two scales and Sa, the number of cells in mm^2^, the medium au, and the Sa vs. the amount of cells in mm^2^ and the medium au. Pearson correlation coefficients were used to measure the correlation between Sa and the amount of cells in mm^2^. We took the following ranges of the r value: r greater than or equal to 0.7—strong correlation; r between 0.7 and 0.5—moderate; and r lower than 0.5—weak correlation. Sample size was calculated on the base of power of test. In this study, we used one-way ANOVA for 5 groups. In this case, the 80% of power of test is achieved for N = 50 in each group.

For texture analysis, between-group comparisons were performed with the one-way ANOVA or the Kruskal–Wallis, test depending on the presence of normal distribution. Statgraphics Centurion version 18.1.12 (StatPoint Technologies Inc., Warrenton, VA, USA) was used for statistical analyses.

## 3. Results

### 3.1. Surface Roughness Outcomes

The Sa average surface height deviation amplitude was highest for the HELT GRADE23 surface and reached 2.66 µm; the lowest value of Sa 0.72 µm was noted for the SLA GRADE23 sample. The surface roughness (Sa) for all the examined surfaces is presented in Table 2.

### 3.2. Biological Analysis Outcomes

#### 3.2.1. In Vitro Cytotoxicity Assessment

**Figure 1:**
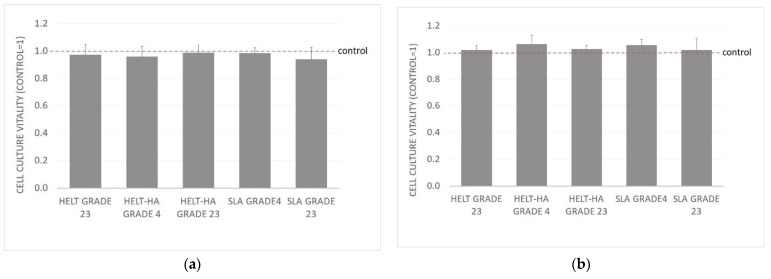
MTT-L929: (**a**) NHDF, (**b**) results after 24 h incubation with the test extracts from the test materials. Results are averages of 5 independent experiments. Results are presented as the ratio of the value obtained in the test to the control culture. There was no statistically significant decrease in the vitality of the culture compared to the control (*p* < 0.05).

The MTT assay evaluated the effect of the tested materials on the vitality of two cell lines. In both cases, no toxicity of the tested biomaterials was found. The results are presented in the graphs (Figure 1). A reference cell line, L929, and a primary human fibroblast line were used in the study. While performing the study, the evaluation of the cell morphology was also performed according to laboratory procedures (identity assessment). The cell morphology was compared with the line specifications and control cultures. No changes in the cell morphology, including the presence of cytopathic changes, were observed in the cultures.

#### 3.2.2. Co-Culture of Cells with Test Materials

In order to exclude direct interaction of the test materials with the cells, co-culture with the cells was also performed. For this purpose, the cells and test materials were placed together on test plates. Again, no changes in the cell morphology or cytopathic effects were observed. In order to confirm the results, a near-field staining was performed to assess the number of live and dead cells. The amount of dead cells in the culture was similar for all test materials and controls. The amount of dead cells was up to 5%.

#### 3.2.3. Cell Attachment

**Figure 2:**
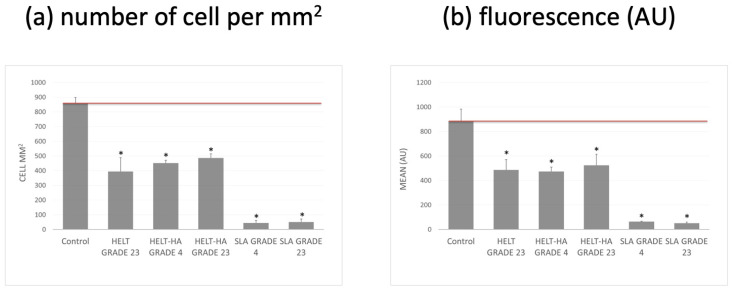
Adhesion of fibroblasts to the tested modified surfaces after 72 h: (**a**) number of cells per mm^2^ of surface tested, (**b**) average fluorescence value measured from an area of 1 mm^2^. * Statistically significant difference (*p* < 0.05) compared to the control.

Another parameter assessed was the adhesion and growth of cells on the tested surfaces. Adherent cells can grow on various surfaces but most often on surfaces made of polystyrene. These are usually subjected to various surface modifications that make the polystyrene surface of the culture vessel more hydrophilic, which ensures maximum adhesion for a wide range of cell types. A good surface that also ensures cell growth is glass, which was also used in the past to make culture dishes. In the tests conducted, the control surface that was optimal for the cells was modified polystyrene (TPP, Trasadingen, Switzerland). The results obtained are presented as the number of cells per test surface and the fluorescence value obtained as the average value from the test surface. The results presented in Figure 2 show that the modifications significantly improved the cell adhesion to the tested surfaces. In the case of the HELT surface, a significant increase in the number of cells is observed, and the degree of adhesion is similar to that of the standard modified polystyrene surface.

The increase in the number of cells per mm^2^ was significantly positively correlated with the increase in the implant surface roughness (Sa), with a strong positive correlation (r = 0.8858).

### 3.3. Fractal Dimension Analysis

Table 3 presents the mean values of the fractal dimension in 100 μm × 100 μm scale. The lowest value of the FD was seen in SLA GRADE4 (1.8047); the highest value was seen in SLA GRADE23 (1.8889). We did not observe significant differences between HELT GRADE23 and HELT-HA GRADE4. HELT-HA GRADE23 and SLA GRADE4 revealed significant differences compared to the other examined surfaces.

Table 4 shows mean values of fractal dimension in 5 μm × 5 μm scale. The lowest value of the FD was seen in SLA GRADE4 (1.6931); the highest value was seen in SLA GRADE23 (1.7732). SLA GRADE4 noted significant differences compared to the other examined surfaces.

Table 5 shows the Pearson correlation coefficient between the FD and Sa and the amount of cells in mm^2^ and medium au. We revealed a strong positive linear correlation (r = 0.92) between Sa and the amount of cells in mm^2^. A weak correlation (r = 0.38) was revealed between the value of the FD (in the scale of 100 μm × 100 μm) and the amount of cells in mm^2^ and medium au. A strong positive correlation (r = 0.73) was seen between FDA and TA in the scale of 100 μm × 100 μm, in contrast to the negative weak correlation in the scale of 5 μm × 5 μm (r = −0.31).

### 3.4. Texture Analysis

**Figure 3:**
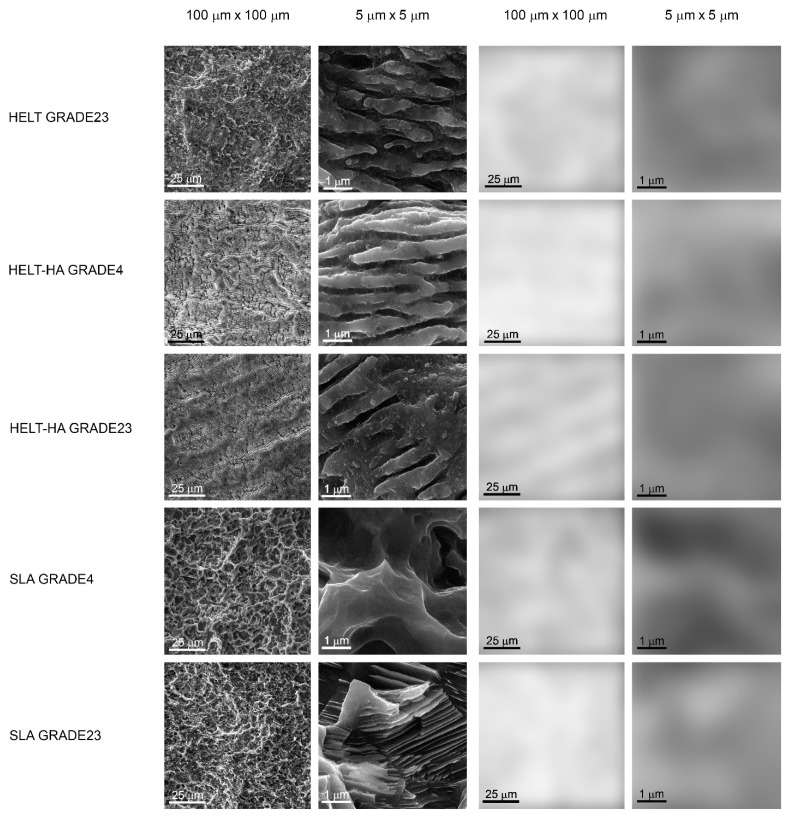
Texture analysis by means of calculation of differential entropy in SEM images in two scales. The two columns on the left show SEM images in the large (100 μm × 100 μm) and small field of view (5 μm × 5 μm). The two columns on the right represent intensity maps of the texture feature studied here in the original SEM image. The whiter areas indicate where the differential entropy is higher (i.e., the surface development is greater, while the darker areas indicate where the differential entropy is low (the implant surface image is more homogeneous)). The differences between the tested surfaces are statistically significantly different from one another (*p* < 0.05) in terms of differential entropy (both at low and high magnification).

An example of the results of investigating the surface structure of the dental implants by SEM image texture analysis is shown in Figure 3.

When examining the differential entropy as a measure of the development of the implant surface, as seen in the SEM, significant differences were noted between the analyzed surfaces in the 100 µm × 100 µm field of view. This texture feature increased as follows (Table 6): SLA_GRADE4 < SLA_GRADE23 < HELT_HA_GRADE4 = HELT_GRADE23 < HELT_HA_GRADE23 ( the symbol “=” means *p* > 0.05, and “<” means *p* < 0.05). Other statistically significant differences are presented in Table 6.

In turn, when examining the differential entropy of the implant surface image at higher magnification (i.e., in a 5 μm × 5 μm field of view), the texture feature increased as follows: SLA_GRADE23 = HELT_HA_GRADE4 = SLA_GRADE4 = HELT_GRADE23 = HELT_HA_GRADE23 (the symbol “=” means *p* > 0.05). More detailed data of the between-group comparisons are presented in Table 7.

The increase in Sa was significantly related with the increase in the differential entropy of the implant surface measured in the 100 *×* 100 µm field of view (Table 8). The value of that texture feature was not proportional and was moderately or strongly related with the fibroblast adhesion.

## 4. Discussion

LIPSS are an arrangement of (quasi) periodic topographic lines representing a linear surface grating structure [17]. There are two kinds of LIPSS, low spatial frequency LIPSS (LSFL, period > λ/2) and high spatial LIPSS (HSFL, period < λ/2), and the period size is close and less than half of the laser wavelength, respectively [18]. A wide range of surface textures can be produced by treating Ti alloys with femtosecond laser radiation and, depending on the processing parameters, ripples, microcolumns, and wavy or smooth surfaces can be obtained [19]. Jörn Bonse [17], in a recent review, has provided a common classification of LIPSS surfaces and has described in detail the possibilities of using this technology in various fields of science.

We found that all the tested samples prepared from Titanium Grade 4 and Grade 23 and modified as described in the materials and methods section revealed no toxicity, and no changes in cell morphology or cytopathic effects were observed. This is a key issue for endosseous titanium implants. Similarly, the recent reports from the literature on the titanium surfaces modified in this way do not indicate any negative properties of the material thus modified. Cunha et al. [20] has reported that the Ti-6Al-4V LIPSS implant surface can potentially improve the osseointegration of titanium dental and orthopedic implants. Schweitzer [21], in a preclinical study, has found that human primary mesenchymal stromal cells (hMSCs) cells cultured on LIPSS surfaces were not compromised in terms of their viability.

In our study, we can observe a greater growth of cells tied together with increased surface roughness, measured as (Sa), and a strong positive correlation (r = 0.8858). We observed a strong correlation of the adhesion and growth of cells on the tested surface with an increase in the implant surface roughness (Sa). SLA Grade 23 sample (Sa = 0.72; cell mm^2^ = 50) was compared to HELT HA grade 23 sample (Sa = 2.20; cell mm^2^ = 486). This seems consistent with the results found in the literature. Wennerberg and Albrektsson [2], in a systematic review concerning the titanium surface topography impact on bone formation, have found that smooth (Sa < 0.5 μm) and minimally rough (Sa 0.5–1 μm) surfaces show less strong bone responses than rougher surfaces, and moderately rough (Sa > 1–2 μm) surfaces show stronger bone responses than rough surfaces (Sa > 2 μm).

Our results show that the HELT and HELT-HA samples exhibit a 10 times greater cell growth per mm^2^ than the SLA samples. Zwahr et al. [22] have used a similar ultrashort pulsed direct laser interference patterning (DLIP) to treat the surface and have found the amount of cells up to 2.5 times higher following 7 days of cultivation medium for the DLIP-structured surface than on the SLA surfaces.

A weak correlation (r = 0.16) between the FD in the 100 μm × 100 μm scale and the Sa parameter was observed, and the r parameter was significantly lower (r = −0.06) for the scale 5 μm × 5 μm. Examination of the correlation between the FD and the physical properties of the material is useful; this was confirmed by Grzebieluch et al. who revealed a strong positive correlation between the FD of composite dental materials and the flexural module [14].

We observed a weak correlation (r = 0.38) between the number of fibroblasts and the fractal dimension in the 100 μm × 100 μm scale. It is interesting that in the 5 μm × 5 μm scale, the correlation coefficient was lower, and it equaled 0.24. Similar results are observed in the case of the correlation between the FD (in both scales) and medium au. It is interesting that the r parameter is relatively higher in the case of the correlation Sa between the number of cells in square mm and medium au. These results were confirmed in a study by Malinowska et al., in which the relations between the fractal dimension of surface of cements and the number of fibroblasts were examined. In the mentioned study, the positive moderate correlation (r = 0.47) between the FD and the amount of fibroblasts was noted, but the examined images were derived from an optical microscope with a lower magnification [23].

Texture analysis can yield deep information about image structure [24]. If the images of implant surfaces are evaluated, the brightness changes observed in the SEM largely represent the surface differentiation. This differentiation can be called the surface roughness or surface development. The texture feature applied here [25] describes the amount of surface development and is directionless [26]. It is simply a measure of the chaos observed in the structure of the surface layer. An image of a machine-polished surface has low entropy, while an image of a sand blasted and etched surface has high entropy, because in the structure of the SEM image there will appear small objects which can be counted [16]. A certain problem is the presence of noise in the analyzed image (e.g., reflected and scattered electrons in the SEM), which can add redundancy and pseudo-data to the calculations [27]. For this reason, it is worth resigning from the full optical depth available in the source images and reducing the number of grey levels to a 6- or 7-bit depth [28]. In this study, ROI was standardized to align the brightness of the compared images to avoid degradation of part of the image texture or differences in the entropy of the structure of the observed surfaces resulting from differences in brightness [26]. Thus, the prepared and analyzed SEM images showing the surface structure indicated that the SLA_GRADE23, HELT_HA_GRADE4, HELT_GRADE23, HELT_HA_GRADE23 (in the large field of view, i.e., 100 µm × 100 µm), and HELT_HA_GRADE23 (in the small field of view, i.e., 5 µm × 5 µm) samples have the most developed surfaces. Therefore, it seems that HELT_HA_GRADE23 would be the best material and surface, especially considering that for the large field of view the result is directly proportional to the roughness measurement (r = 0.67; *p* < 0.05). It is interesting that the value of the FD and DifEntrp is strongly positively correlated (r = 0.73) in the scale of 100 μm × 100 μm, in contrast to the weak negative correlation (r = −0.31) in the scale of 5 μm × 5 μm.

## 5. Study Limitations

The limitation of the study is the lack of sample chemical composition and wettability. Further research on these experimental surfaces is planned.

## 6. Conclusions

- The present work shows that surface nanotexture laser LIPSS did not affect the sample cytotoxicity. A greater cell growth of NHDF cells was observed on the LIPSS samples.
- Texture and fractal dimension analyses are promising methods for evaluating dental implants with complex geometry. Moreover, when applied in the evaluation of a new implant surface at the physical level, the prediction of cell behavior can be achieved. This allows for the averting of time-consuming and cost-elevating cell tests of non-promising new implant surfaces.
- Texture and fractal dimension analyses are promising methods for evaluating dental implants with complex geometries, and further studies should be carried out to make them more useful for wider applications

## Figures and Tables

**Table 1:** Preparation of the titanium plates.

No	Name	Titanium Grade	Method of Preparation
1	SLA GRADE4	Grade 4	Al_2_O_3_ sandblasting process with the fraction of 30–100 µm. Purified samples were subjected to the etching process (conditions: oxalic acid 100 g/L, time: 60 min, temperature: boiling). Samples washed in an ultrasonic cleaner (DEMI water, time: 10 min).
2	SLA GRADE23	Grade 23	
3	HELT GRADE 4	Grade 4	LIPSS-based Low Spatial Frequency LIPSS (LSFL) surface treated with a subpicosecond pulse laser with variable energy in 5 and 50 µJ. With the focal spot of 50 µm the F_tot_ was kept below 0.8 J/cm^2^. Beam spot overlap > 80%. Samples washed in an ultrasonic cleaner (DEMI water, time: 5 min).
4	HELT GRADE23	Grade 23	
5	HELT-HA GRADE4	Grade 4	LIPSS-based Low Spatial Frequency LIPSS (LSFL) surface treated with a subpicosecond pulse laser with variable energy in 5 and 50 µJ. With the focal spot of 50 µm the F_tot_ was kept below 0.4 J/cm^2^. Next, Hydroxyapatite (50 g HA solution/100 mL C_2_H_5_OH solution) applied by immersion method; this operation was repeated three times. Samples washed in an ultrasonic cleaner (DEMI water, time: 5 min).

**Table 2:** Mean values of surface roughness (Sa) for each examined surface in µm (SD—standard deviation).

Surface Name	Sa	SD
HELT GRADE23	2.66	0.07
HELT-HA GRADE4	2.43	0.07
HELT HA GRADE23	2.20	0.09
SLA GRADE4	1.42	0.02
SLA GRADE23	0.72	0.01

**Table 3:** Post hoc ANOVA results (least significant difference) for comparison of fractal dimension (FD) for ROI size 100 μm× 100 μm between each examined surface (SD—standard deviation).

	Surface Name	FD (ROI = 100 μm × 100 μm)		*p* < 0.05
		Mean	SD	
1	HELT GRADE23	1.8812	0.0045	3, 4, 5
2	HELT-HA GRADE4	1.8761	0.0070	3, 4, 5
3	HELT-HA GRADE23	1.8596	0.0125	1, 2, 4, 5
4	SLA GRADE4	1.8047	0.0076	1, 2, 3, 5
5	SLA GRADE23	1.8889	0.0084	2, 3, 4

**Table 4:** Post hoc ANOVA results (least significant difference) for comparison of fractal dimension (FD) for ROI size 5 μm × 5 μm between each examined surface (SD—standard deviation).

	Surface	FD (ROI = 5 μm × 5 μm)		*p* < 0.05
		Mean	SD	
1	HELT GRADE23	1.7315	0.0073	2, 4, 5
2	HELT-HA GRADE4	1.7726	0.0135	1, 3, 4
3	HELT-HA GRADE23	1.7364	0.0101	2, 4, 5
4	SLA GRADE4	1.6931	0.0061	1, 2, 3, 5
5	SLA GRADE23	1.7732	0.0166	1, 3, 4

**Table 5:** The values of the Pearson correlation coefficient (r) between the value of fractal dimension (FD) calculated in different scales (100 μm × 100 μm and 5 μm × 5 μm) and the Sa, number of cells per mm^2^ and medium au, and differential entropy (DifEntrp).

Feature	vs.	Feature	R
FD (100 μm × 100 μm)	vs.	Sa	0.16
FD (5 μm × 5 μm)	vs.	Sa	−0.06
FD (100 μm ×100 μm)	vs.	cells [mm^2^]	0.38
FD (5 μm × 5 μm)	vs.	cells [mm^2^]	0.24
FD (100 μm ×100 μm)	vs.	medium au	0.38
FD (5 μm × 5 μm)	vs.	medium au	0.19
Sa	vs.	cells [mm^2^]	0.86
Sa	vs.	medium au	0.92
FD (100 mm × 100 mm)		DifEntrp (100 µm×100 µm)	0.73
FD (5 mm × 5 mm)		DifEntrp (5 µm × 5 µm)	−0.31

**Table 6:** Post hoc ANOVA results (least significant difference) for comparison of differential entropy (DifEntrp) for ROI size 100 μm × 100 μm between each examined surface (SD—standard deviation).

	Surface	DifEntrp		*p* < 0.05
		Mean	SD	
1	HELT GRADE23	1.3602	0.0152	3, 4, 5
2	HELT-HA GRADE4	1.3501	0.0192	3, 4, 7
3	HELT-HA GRADE23	1.3884	0.0112	1, 2, 4, 5
4	SLA GRADE4	1.2504	0.0062	1, 2, 3, 5
5	SLA GRADE23	1.3252	0.0055	1, 2, 3, 4

**Table 7:** Post hoc ANOVA results (least significant difference) for comparison of differential entropy (DifEntrp) for ROI size 5 μm × 5 μm between each examined surface (SD—standard deviation).

	Surface	DifEntrp		*p* < 0.05
		Mean	SD	
1	HELT GRADE23	1.1741	0.0296	5
2	HELT-HA GRADE4	1.1199	0.0326	3
3	HELT-HA GRADE23	1.1884	0.0496	2.5
4	SLA GRADE4	1.1180	0.0738	
5	SLA GRADE23	1.0897	0.0286	1.3

**Table 8:** The values of the Pearson correlation coefficient (r) between the value of differential entropy (DifEntrp) calculated in different scales (100 μm × 100 μm and 5 μm × 5 μm) and the Sa, the number of cells per mm^2^, and medium au. No statistically significant relations were found.

Feature	vs.	Feature	R
DifEntrp (100 µm × 100 µm)	vs.	Sa	0.56
DifEntrp (5 µm × 5 µm)	vs.	Sa	0.75
DifEntrp (100 µm × 100 µm)	vs.	cells [mm^2^]	0.84
DifEntrp (5 µm × 5 µm)	vs.	cells [mm^2^]	0.75
DifEntrp (100 µm × 100 µm)	vs.	medium au	0.83
DifEntrp (5 µm × 5 µm)	vs.	medium au	0.79

## Data Availability

Data available on request.
